# A Text-Book of Surgery

**Published:** 1887-04

**Authors:** 


### Book Reviews.

---

A Text-Book on Surgery, General, Operative and Mechanical. By John A. Wyeth, M. D., Professor of Surgery in the New York Polyclinic; Surgeon to Mount Sinai Hospital; Consulting Surgeon to the Yorkville Dispensary and Hospital for Women and Children; to the Women's Hospital of Brooklyn; Ex-President of the New York Pathological Society; Member of the New York Surgical Society; of the Academy of Medicine; of the New York State Medical Association; of the New York County Medical Society; Honorary Member of the Texas State Medical Society; of the College of Physicians and Surgeons, Little Rock, Arkansas; author of an Essay on the Surgical Anatomy of the Tibio-torsal Region, with special reference to amputation at this joint; awarded the James R. Wood Annual Prize of the Bellevue Alumni Association, 1876; An Essay on the Surgical Anatomy and History of the Carotid Arteries, awarded the First Prize of the American Medical Association, 1878; An Essay on the Surgical Anatomy and History of the Innominate and Subclavian Arteries; awarded the Second Prize of the American Medical Association, 1878, etc. New York: D. Appleton & Company, 1887.

The first part of this book is devoted to a description of the methods of preparing and preserving the apparatus needed for dressing wounds in the antiseptic practice of to-day. The author says the conditions which would justify the application of a silk, metal or any non-absorbable ligature to an artery are rarely present. He advises the cat-gut ligature, and gives minute description for their proper preparation and preservation. He says silk is invaluable for sutures, but should not be used for ligatures except in certain operations within the abdominal cavity, or in wounds which are to be treated by the open method.

The chapter on bandaging is full and complete, embracing as it does most perfect illustrations and descriptions of the various bandages and their application.

Anæsthesia is the subject of the third chapter. Here we have the various agents explained. For local anæsthesia the author's preference is given to cocaine, a four percent. solution being the strength usually employed by him. For general anæsthesia, he says of the various anæsthetics which have been introduced for surgical use, only two deserve to be considered, and in order of preference they are ether and chloroform. Ether he regards as much safer.

The chapters on amputations, ligation of arteries and hernia are especially valuable on account of the excellent illustrations, which are superior to any we ever saw, and they can but prove invaluable to the operator.

The work contains 777 pages, and covers the entire field of general surgery, every part of it bearing evidences of having been written by a master.

